# Accessing invasion-competent Plasmodium vivax merozoites reveals antibody-dependent NK cell activation

**DOI:** 10.21203/rs.3.rs-9011486/v1

**Published:** 2026-04-01

**Authors:** Jeremy Salvador, Lucas Guselli, Romminea Yeat, Sokchea Lay, Titsophantharoth Thol, Nisa Ya, Lionel Feufack-Donfack, Sievleang Heng, Candice Bohaud, Agnes Orban, Léa Baldor, Baura Tat, Chetan Chitnis, Tineke Cantaert, Jean Popovici

**Affiliations:** Institut Pasteur du Cambodge; Institut Pasteur du Cambodge; Institut Pasteur du Cambodge; Institut Pasteur du Cambodge; Institut Pasteur du Cambodge; Institut Pasteur du Cambodge; Institut Pasteur du Cambodge; Institut Pasteur du Cambodge; Institut Pasteur du Cambodge; Institut Pasteur du Cambodge; Institut Pasteur du Cambodge; Institut Pasteur du Cambodge; Institut Pasteur; Institut Pasteur du Cambodge; Institut Pasteur du Cambodge

## Abstract

Malaria pathogenesis and clinical symptoms arise from blood-stage replication of Plasmodium parasites, which depends on rapid invasion of red blood cell by merozoites. Despite the central importance of this stage, direct functional studies of *Plasmodium vivax* merozoites have been severely limited by the lack of continuous *in vitro* culture and the inability to routinely access viable, invasion-competent merozoites from clinical isolates. Consequently, key aspects of *P. vivax* merozoite biology and immune recognition remain poorly defined.

Here, we establish a robust platform that enables synchronized release and high-yield isolation of invasion-competent *P. vivax* merozoites directly from *ex vivo* cultures. Reversible inhibition of parasite egress using the cGMP-dependent protein kinase inhibitor ML10 allows precise synchronization at the segmented schizont stage, followed by rapid mechanical liberation of merozoites using a liposome-based extrusion approach. This workflow yields highly pure merozoites with recovery rates exceeding 75% while preserving structural integrity and invasive capacity.

Leveraging this platform, we provide direct functional evidence that antibody opsonized *P. vivax* merozoites activate human natural killer cells consistent with antibody-dependent cellular cytotoxicity mechanisms. Together, this study overcomes a long-standing technical barrier and enables direct investigation of *P. vivax* merozoite invasion biology and immune effector responses.

## Introduction

Malaria is a mosquito-borne parasitic disease and remains a major public health concern, causing hundreds of thousands of deaths each year. According to the World Health Organization, an estimated 263 million cases of malaria were reported worldwide in 2023, primarily in Africa, Asia, and Latin America^1^. Symptoms typically appear within two weeks after infection and result from *Plasmodium* parasite replication within red blood cells (RBCs)^2^. This stage begins when the merozoite, the invasive form responsible for the blood stage of infection, actively invades the RBC. The erythrocytic cycle comprises successive stages: ring, trophozoite, and schizont. After asexual replication at the schizont stage, the parasite undergoes schizont segmentation, ruptures the host cell, and releases new merozoites capable of invading other RBCs. This process leads to the cyclical destruction of the infected red blood cells and the clinical symptoms of malaria^2^.

Schizont rupture primarily depends on calcium signaling and cyclic GMP-dependent protein kinase (PKG) activity in *Plasmodium* spp^3, 4^. PKG is highly conserved among *Plasmodium* species and plays a crucial role by triggering the release of exonemes and micronemes containing proteins essential for disrupting the parasitophorous vacuole and erythrocyte membranes^4, 5, 6, 7^. Tight regulation of this signaling cascade is therefore critical for coordinated parasite egress and subsequent merozoite release.

Once released, merozoites remain extracellular for only a short period (from seconds to minutes^8, 9^) during which they are transiently exposed to the host immune system. This brief extracellular stage represents a key target for immune responses and antimalarial interventions. Blocking RBC invasion through drugs or vaccines targeting merozoites or specific surface antigens could halt the asexual cycle, reduce parasite burden, and prevent the onset of malaria symptoms. Accordingly, obtaining viable merozoites is critical not only to dissect invasion mechanisms but also to evaluate functional antibody-mediated and innate effector responses against this parasite stage.

Merozoite invasion of red blood cells is a highly orchestrated process governed by conserved molecular mechanisms across *Plasmodium* species. However, significant differences between species in parasite ligand/host receptor interactions have been documented^10^. Detailed characterization of these differences has been limited by the difficulty of adapting most human malaria parasites to *in vitro* culture. The notable exception is *P. falciparum*, which was successfully cultured in the 1970s^11^. In contrast, attempts to culture-adapt *P. vivax*, the most globally widespread human malaria parasite, have largely failed, partly due to its strict preference for the rare population of circulating immature reticulocytes^12, 13^. Consequently, precise elucidation of the cellular and molecular distinctions between species has progressed only gradually.

Although progress has been made with the culture adaptation of *P. knowlesi*, a simian *Plasmodium* species closely related to *P. vivax* and causing zoonotic malaria in Southeast Asia, direct *in vitro* studies on *P. vivax* remain extremely challenging because it cannot be amplified in culture^13, 14^. The limited parasite numbers available severely constrain the application of protocols developed for *P. falciparum* or *P. knowlesi* to *P. vivax*. The study of the merozoite stage is further complicated by the fragility of merozoites and the difficulty of purifying sufficient viable cells for downstream experiments. While several protocols have been developed to isolate merozoites from *P. falciparum* and *P. knowlesi*, these approaches are often limited by low yield, poor purity, or prolonged harvesting periods (2–3 hours), which can compromise cell viability^8, 15, 16, 17^. To date, none of these methods have enabled routine recovery of viable *P. vivax* merozoites in quantities and quality sufficient for functional and immunological analyses.

Here, we address a major technical barrier in *P. vivax* research by establishing a robust and scalable workflow to routinely isolate large numbers of highly pure and viable *P. vivax* (and *P. falciparum*) merozoites. We demonstrate that reversible inhibition of PKG using the specific inhibitor ML10 enables synchronization at the segmented schizont stage, followed by mechanical release of invasion-competent merozoites using a liposome extrusion approach. This strategy yields viable merozoites capable of invading new red blood cells, functional investigation of merozoite biology and immune effector interactions that were previously inaccessible for *P. vivax*.

## Materials and Methods

### Patient collections.

*P. vivax* -infected blood used in this work was obtained from patients seeking treatment in collaborating local health facilities of Stung Treng and Kampong Speu provinces in Cambodia. Patients positive to *P. vivax* by rapid diagnostic test were offered to participate. Written informed consent was obtained prior to inclusion in the study. Upon consent, a venous blood sample was collected in heparin tubes prior to antimalarial treatment provided by the local health care providers following national treatment guidelines of Cambodia. Blood from *P. vivax* seropositive individuals was obtained from an ongoing longitudinal cohort study conducted in malaria-endemic area of Eastern Cambodia. Ethical approval was obtained from the National Ethics Committee for Health Research of Cambodia (NECHR nr 2025 − 297)

Blood from healthy donors was obtained from accompanying adults visiting the laboratory of Medical Biology, Institut Pasteur du Cambodge (NECHR nr 2024–004). Donors self-declared healthy based on case report form.

### Blood sample collection and processing.

Blood was immediately either shipped to our laboratory in Phnom Penh or cryopreserved on site using glycerolyte 57 solution (Baxter, USA) as previously described^18, 19^. *P. vivax* mono-infection was verified by PCR using established protocols^20, 21, 22^. Whether used fresh or after cryopreservation, blood was first centrifuged to remove plasma and washed with RPMI and leukocytes were depleted using NWF filters^23^. Cryopreserved samples were thawed using step-wise NaCL solutions as previously described^19^. After thawing or upon leukocyte depletion for fresh samples, RBC pellets were resuspended in complete Iscove’s Modified Dulbecco’s Medium (cIMDM: IMDM supplemented with 5% malaria-naïve human AB serum, 2% hypoxanthine, 0.2% glucose and 0.5% Albumax 0.5%) at 25% hematocrit. Parasites were enriched using a Percoll-KCl density gradient as previously described^24^. Briefly, a Percoll-KCL solution was prepared at 57.5% and the infected blood was carefully layered at the surface of the Percoll-KCL solution. After centrifugation at 1,200 RCF for 15 minutes at room temperature, infected red blood cells were collected and washed three times with RPMI. Infected blood was then resuspended in cIMDM at 2–5% hematocrit and incubated for short-term culture under 5% CO2, 5% O2 atmosphere at 37°C.

Plasma was obtained after centrifugation and immediately stored at −80°C. After thawing, plasma was heat-inactivated (56°C for 30 minutes) before usage.

### In vitro culture of P. falciparum 3D7 strain.

Cultures were maintained in RPMI-1640 medium supplemented with 0.5% Albumax, 25 mM HEPES, 0.1 mM hypoxanthine, 50 μg/mL gentamicin, and human plasma, under 5% CO2, 5% O2 atmosphere at 37°C. To synchronize the culture, regular sorbitol treatment was performed to enrich ring-stage parasite^25^. ML10 was used before merozoites isolation for 6 hours at 25 nM^26^.

### Parasite monitoring.

Parasite monitoring was carried out by Giemsa stain thin or thick smears. Every smear was done with a minimum of 0.3 μL of infected blood pellet, smears were fixed with methanol and stained with 5% Giemsa during 5 minutes for thin smear or with 10% Giemsa during 10 minutes for thick smear. Two independent, blind observers counted 20,000 RBCs per smear across randomly selected fields, classifying each infected cell as ring, trophozoite, non-segmented schizont, segmented schizont and dead schizont based on standard morphology. Stage ratios were calculated as the count of each stage divided by the total number of infected RBCs.

### Plasmodium vivax ML10 treatment.

**Figure 1:**
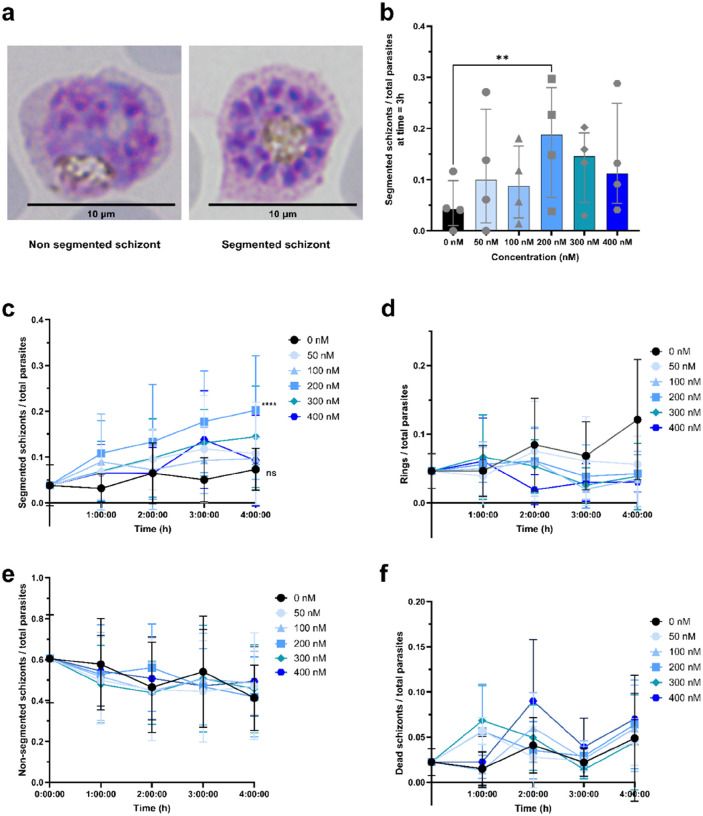
Synchronization of *Plasmodium vivax* cultures at the segmented schizont stage. **a,**Giemsa-stained thin blood smears showing a mid-schizont initiating segmentation (left) and a fully segmented schizont (right) from *P. vivax ex vivo* cultures (5% Giemsa). **b,**Proportion of segmented schizonts relative to total parasites (live and dead) after 3 h of ML10 treatment at increasing concentrations. **c–f,**Proportions of segmented schizonts (**c**), ring-stage parasites (**d**), non-segmented schizonts (**e**), and dead schizonts (**f**) relative to total parasitemia (live and dead) in *P. vivax ex vivo* cultures treated or not with ML10 at different concentrations over a 4 h period. (b) Data are shown as median with interquartile range (IQR). Statistical analyses were performed using aFriedman test followed by Dunn’s multiple comparisons test (**p < 0.01). (c) Data are shown as mean ± SD. Statistical analysis was performed using, a two-way repeated-measures ANOVA (concentration × time) followed by Dunnett’s multiple comparisons test with a single pooled variance. For clarity, statistical annotations are shown only at 4 h and refer to the comparison between 200 nM ML10 and the 0 nM control at this time point. ****p < 0.0001; ns, not significant.

The *in vitro P. vivax* cultures were regularly monitored by Giemsa smear and the ML10 treatment was started when the majority of parasites reach the late stage schizonts (Fig. 1a). Parasites were treated with different concentrations of ML10, 0nM, 50 nM, 100 nM, 200 nM, 300 nM, and 400 nM during 0h, 1h, 2h, 3h, and 4h to identify the optimal concentration and exposure time for blocking egress without affecting parasite integrity.

### C-chip counting.

To quantify uninfected RBCs, infected RBCs, and isolated *P. falciparum* and *P. vivax* merozoites, samples were incubated 15 minutes prior to counting with Hoechst at 1 μg/mL and washed 3 times in cIMDM + 200nM of ML10. Samples were then loaded into a hemocytometer chamber (SKC, Inc. C-Chip^™^) and observed at 40x using a fluorescence microscope equipped with a camera. Images were captured in both brightfield and fluorescence, Hoechst dye was excited with a laser at 350 nm and observed with a 460 nm filter, allowing visualization of parasite nuclei within infected cells or isolated merozoites.

### P. vivax PFA fixation.

Segmented *P. vivax* schizonts were fixed using a 4% paraformaldehyde (PFA) solution. Parasites were centrifuged for 1 min in a benchtop centrifuge, and the supernatant was removed. The pellet was then resuspended in PBS–4% PFA (500 μL per 1–20 μL of pellet) or in PBS–4% PFA supplemented with 0.2% Hoechst, and incubated for 15 min at room temperature, protected from light. The pellet was subsequently washed four times with 1 mL of PBS and finally resuspended in 300 μL to 1 mL of PBS prior to isolation using a liposome extruder. Following fixation and during the final washing steps, non-infected red blood cells were observed to disappear, whereas parasites remained intact.

Fixed parasites were either stored at 4°C for up to one week before extrusion or cryopreserved in PBS supplemented with 5% trehalose, using a volume corresponding to 5–10 times the remaining pellet. Samples were placed in a controlled-rate freezing container and stored at − 80°C.

### Mechanical disruption of schizonts and merozoite isolation.

To isolate merozoites, a liposome extruder with a polycarbonate membrane filter of 2 μm was used (HandExtruder^™^ 1 mL, Genizer LLC, California, USA). Determination of the optimal number of filtration cycles (FC) required to rupture the RBC membrane without damaging the merozoites was conducted on *P. falciparum* 3D7. One FC consists of one back-and-forth passage of the parasite solution through the filter, followed by one additional passage through the filter. The additional passage through the filter allows collection of the merozoite sample on the opposite side of the initial compartment, thereby ensuring that all recovered parasites have passed through the filter at least once. *P. falciparum* infected RBCs were resuspended in 1–5% hematocrit in PBS 1X and processed with 5, 10, 15, 20 and 25 FC. The efficiency of the extrusion was quantified on a hemocytometer chamber (SKC, Inc. C-Chip^™^) slide using fluorescent microscopy and image J software. Based on *P. falciparum* results, *P. vivax* merozoites isolation was performed in IMDM or PBS 1X at 1–5% hematocrit, using a 2 μm polycarbonate membrane filter processed with 10 FC. For PFA-fixed parasites, 15 FC were required to achieve improved merozoite isolation while minimizing aggregation.

### Immunofluorescence assay.

The presence of isolated merozoites was confirmed by an immunofluorescence assay targeting the surface protein PvDBP (*Plasmodium vivax* Duffy Binding Protein). IFA slides were coated with Poly-L-lysine, followed by a final rinse with distilled water. Merozoites or schizont suspension were applied on the slide and fixed with acetone (90%) / methanol (10%) at −20°C for 10 minutes. A rabbit polyclonal anti-PvDBP antibody^27^ was used at 50 μg/mL in PBS-BSA 1% for 1h30 at RT, followed by three consecutive washes of 5 minutes with PBS − 1% BSA -Tween 0.05%. Secondary anti-rabbit conjugated with the AlexaFluor488 at 2 μg/mL, and Hoechst dye at 1 μg/mL in PBS-BSA 1% were added for 30 minutes at RT followed by 3 washes of 5 minutes with PBS on shaking plate. Slides were then observed using fluorescent microscope Leica Dm2500, images were captured and then processed using ImageJ software. Negative controls followed the same protocol except using PBS instead of primary antibody.

### Quantification of merozoite yield.

To assess the efficiency of merozoite recovery, the theoretical number of merozoites after filtration was estimated based on the number of segmented schizonts prior to extrusion multiplied by the average number of merozoites per segmented schizont. The theoretical number of merozoites was calculated using the following formula:

Theoretical number of merozoites=(segmented schizont parasitemia before extrusion)×(total number of cells[iRBCs+RBCs])×(average number of merozoites per schizont,estimated from Giemsa-stained smears).

Segmented schizont parasitemia was determined from Giemsa-stained smears prior to extrusion, and the total number of iRBCs and RBCs was determined using a C-Chip counting hemocytometer. The recovery rate was then expressed as the percentage of merozoites recovered relative to the theoretical estimate, calculated as follows:

Recovery rate(%)=(number of merozoites recovered after extrusion×100)/(theoretical number of merozoites).

### Invasion assay.

Invasion assays were performed using isolated *P. falciparum* and *P. vivax* merozoites. For each assay, schizont-stage parasites were directly resuspended in IMDM medium and subjected to 10 FC using a liposome extruder equipped with a 2-μm polycarbonate membrane. Immediately before merozoite isolation, 96-well plates were prepared with 5 × 10^6^ red blood cells (RBCs) or reticulocytes (for *P. vivax* assays) in a total volume of 5 μL of IMDM per well. The freshly isolated merozoite suspension was then added directly to each well at a 1:4 merozoite-to-RBC ratio. The merozoite concentration used for calculating the required volume was based on the theoretical number of merozoites determined prior to isolation. The final volume in each well was adjusted to 100 μL with IMDM medium. After 1 hour of incubation, 5% human AB plasma was added to the culture to support parasite invasion and development. Parasite invasion and subsequent development were monitored by Giemsa-stained thin smears at 1 h, 2 h, 5 h, 24 h, and 48 h post-incubation.

### Optical microscopy and image processing.

Giemsa-stained thin blood smears and fresh Giemsa preparations were imaged using a light microscope equipped with a 100× objective and an Olympus DP72 camera. Images were subsequently processed using ImageJ (version X) for adjustment of brightness and contrast. To improve image clarity, the following filters were applied uniformly to all images: Gaussian blur (radius = 1.5), median filter (radius = 2), and unsharp mask (radius = 2, mask = 0.6).

### Isolation of human NK cells.

Peripheral blood mononuclear cells (PBMCs) were isolated from fresh whole blood obtained from healthy adult donors by density gradient centrifugation using Ficoll-Paque. NK cells were purified by negative selection using a commercial human NK cell isolation kit according to the manufacturer’s instructions (Miltenyi). Isolated NK cells were resuspended in complete RPMI medium supplemented with 10% heat-inactivated fetal bovine serum (FBS), L-glutamine, and penicillin streptomycin antibiotics, and were used on the day of isolation for functional assays.

### NK cell stimulation and co-culture conditions.

Purified merozoites were incubated with heat-inactivated plasma obtained from either healthy donors (HDs) or *P. vivax*-seropositive individual at 37°C for 60 minutes.

Purified NK cells were incubated with anti-human CD107a APC antibody (clone: H4A3, Biolegend) and Monensin (dilution 1:30, Biolegend) for 5 minutes at room temperature. NK cells were then co-cultured with opsonized *P. vivax* merozoites at a defined effector-to-target ratio (NK:merozoite ratio of 1:2) in V-bottom 96-well plates. Co-cultures were incubated for 6 hours at 37°C with 5% CO_2_. Control conditions included (i) NK cells cultured with merozoites pre-incubated with healthy donor plasma, (ii) NK cells cultured with merozoites in the absence of plasma, and.

### Flow cytometric analysis of NK cell activation.

After incubation, NK cells were washed and stained with Zombie Aqua^™^ Fixable Viability Kit (Biolegend) for distinguishing live and dead cells followed by surface staining with following anti-human antibodies: anti-CD56 BV421 (clone: 5.1H11, Biolegend), anti-CD69 PE-Cy7 (clone: FN50, Biolegend) and anti-CD3 PerCp Cy5.5 (clone: OKT3, Biolegend). Cells were fixed and permeabilized with True-Nuclear^™^ Transcription Factor Buffer Set (Biolegend) as per kit instructions and stained intracellularly with human anti-IFNγ PE antibody (clone: B27, Biolegend). Samples were acquired on a FACSAria Fusion (BD Biosciences) and data analyzed using FlowJo version 10.0. Detailed gating strategies are shown in Fig. 4a.

### Statistical analysis.

Statistical analyses were performed using GraphPad Prism (version 10.6.1). Data are presented as mean ± SD or median with interquartile range (IQR), as indicated. The statistical tests used, sample sizes, and definitions of significance are specified in the figure legends. Unless otherwise stated, all tests were two-tailed and a P value < 0.05 was considered statistically significant.

## Results

### ML10-mediated synchronization of P. vivax cultures at the segmented schizont stage.

To investigate the effect of ML10 on *P. vivax* egress, *in vitro* cultures were established from cryopreserved isolates. Parasite development was routinely monitored by Giemsa-stained thin smears. ML10 treatment was initiated once the majority of parasites had reached the non-segmented schizonts stage (Fig. 1a). Following exposure to varying concentrations and durations of ML10, the proportions of segmented schizonts, ring-stage parasites, non-segmented schizonts, and dead schizonts were quantified by Giemsa-stained smears (Fig. 1b–f).

A significant increase in the proportion of segmented schizonts was observed after 3 h of incubation with 200 nM ML10, with the median increasing from 0.043% (IQR 0.010–0.098) to 0.1875% (IQR 0.065–0.2795). This corresponds to 4.4-fold increases relative to 0 nM ML10 (p < 0.05) (Fig. 1b). More generally, after 1 h of ML10 exposure, all treated cultures displayed a higher proportion of segmented schizonts compared with the untreated control. Over the 4 h treatment period, cultures exposed to 50 nM, 100 nM, 200 nM, and 300 nM ML10 showed a progressive accumulation of segmented schizonts. Treatment with 200 nM ML10 at 4 h yielded the maximal proportion of segmented schizonts observed (0.202% ± 0.119, mean ± SD), significantly higher than the control condition (0.073% ± 0.046, mean ± SD). (Fig. 1c).

Consistent with these findings, in untreated cultures the proportion of ring-stage parasites increased over the 4 h period due to egress and reinvasion, whereas it remained stable in ML10-treated cultures (Fig. 1d). Concomitantly, the proportion of non-segmented schizonts declined progressively in both control and ML10-treated cultures (Fig. 1e), confirming that non-segmented schizonts continued to develop into segmented forms in the presence of ML10. Notably, no significant increase in the proportion of dead schizonts was detected in ML10-treated cultures compared with the control (Fig. 1f).

The ML10-mediated block of egress was reversible, as removal of the compound restored schizont rupture and merozoite release. This was directly visualized by live imaging, showing synchronized schizont bursting following ML10 washout (Supplementary Video 1), consistent with previous observations in *P. falciparum* and *P. knowlesi*.

Together, these data demonstrate that ML10 effectively and reversibly delays or blocks egress of *P. vivax* for at least 4 h, indicating that segmented schizonts continue to accumulate over the incubation period. Based on these results, we established 200 nM ML10 applied for 3–4 h as the standard condition to synchronize *P. vivax* at the segmented schizont stage. These optimized parameters were subsequently used in downstream experiments throughout this study.

### High-purity, high-yield isolation of viable Plasmodium falciparum merozoites by manual filtration using a liposome extruder.

To establish the optimal experimental conditions for merozoite isolation using the liposome extruder system, we first performed a series of assays with *P. falciparum* 3D7 strain. Parasite cultures were synchronized successively with sorbitol and ML10 to obtain tightly synchronized segmented schizonts. After 3 hours of ML10 incubation, parasites were stained with Hoechst, enabling visualization of merozoite nuclei following fixation.

**Figure 2:**
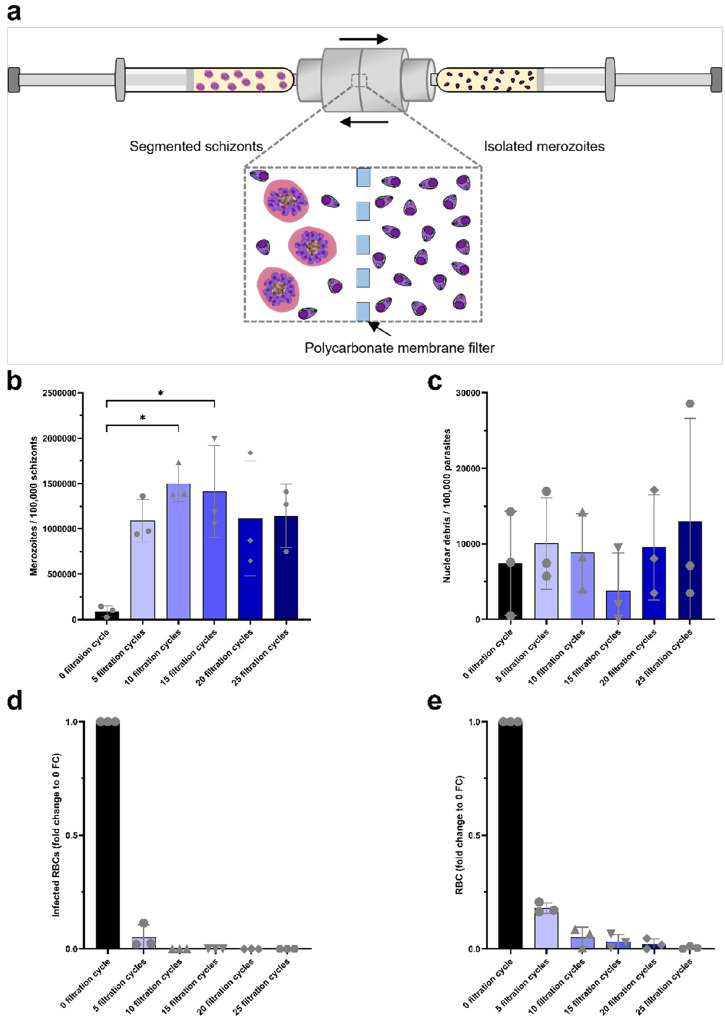
Mechanical release of *Plasmodium falciparum* merozoites using a manual liposome extruder. **a,**Schematic representation of the operating principle of the liposome extruder used for the mechanical release of merozoites from segmented *P. falciparum* schizonts. **b,** Number of merozoites recovered after 0, 5, 10, 15, 20 and 25 filtration cycles (FC), expressed per 100,000 segmented schizonts counted prior to filtration. **c,** Number of nuclear debris detected after 0, 5, 10, 15, 20 and 25 filtration cycles (FC), expressed per 100,000 segmented schizonts counted prior to filtration. **d,** Number of *P. falciparum*–infected red blood cells (iRBCs) remaining after 0, 5, 10, 15, 20 and 25 filtration cycles (FC), expressed per 100,000 segmented schizonts counted prior to filtration. **e,** Number of uninfected red blood cells remaining after 0, 5, 10, 15, 20 and 25 filtration cycles (FC), expressed per 100,000 segmented schizonts counted prior to filtration. Data are shown as median with range (minimum to maximum). Paired statistical comparisons were performed using a Friedman test followed by Dunn’s multiple comparisons test.

Synchronized *P. falciparum* cultures were then subjected to successive filtration cycles (FC) in order to determine the optimal conditions (number of FC and membrane size) for efficient merozoite recovery (Fig. 2a).

Following each FC, the number of merozoites, nuclear debris, intact infected RBCs, and RBCs were quantified microscopically using a C-Chip hemocytometer. Merozoites were distinguished from debris and intact schizonts based on morphology and the presence of fluorescent nuclei, as determined from the overlay of brightfield and fluorescence images analyzed using ImageJ (Supplementary Fig. S1). These results are also consistent with observations obtained from Giemsa-stained thin smears after 0, 1, 5, 10, 15, 20, and 25 FC (Supplementary Fig. S2a).

Merozoite yield, expressed per 100,000 initial schizonts, increased from 90,900 ± 60,200 (mean ± SD) at 0 FC to a maximum of 1.5 × 10^6^ ± 199,900 (mean ± SD) after 10 FC, corresponding to a 17.5-fold increase (Fig. 2b). Beyond 10 FC, merozoite yield tends to progressively decline, indicating that excessive filtration could adversely affect merozoite recovery.

Quantification of nuclear debris per 100,000 initial schizonts did not reveal a significant increase in nuclear debris compared with the unfiltered culture (Fig. 2c).

Filtration was also highly effective at removing both infected and uninfected red blood cells. The proportion of infected RBCs decreased to 5% after 5 FC and was completely eliminated by 10 FC (Fig. 2d). Similarly, uninfected RBCs were reduced by 82.0%, 94.4%, 95.0%, 96.7%, and 98.0% after 5, 10, 15, 20, and 25 FC, respectively (Fig. 2e)

After 10 FC, we achieved a mean recovery rate of 75.15 ± 9.99% (mean ± SD), demonstrating the high efficiency and reproducibility of the liposome extruder–based approach for isolating viable *P. falciparum* merozoites (Fig. 2b).

We also evaluated the use of a 5 μm membrane filter. Although free merozoites were detected after five FC, this condition resulted in a high proportion of infected red blood cells as well as parasites released from their host cells while retaining an intact parasitophorous vacuole (Supplementary Fig. S2b).

To assess the viability and invasive capacity of merozoites obtained after mechanical filtration, invasion assays were performed by adding fresh red blood cells (RBCs) to the merozoite suspension collected after 10 FC. Prior to the assay, the absence of residual infected RBCs in the merozoite preparation was confirmed by Giemsa-stained smear analysis, ensuring that subsequent infections originated exclusively from newly invaded cells.

After 3 hours of incubation, Giemsa-stained smears revealed the presence of *P. falciparum* ring-stage parasites, indicating that the isolated merozoites retained their ability to invade erythrocytes (Supplementary Fig. S3a). Following 24 hours of culture, parasites exhibited normal intraerythrocytic development and had reached trophozoite/early schizont stage, confirming that the parasites remained developmentally competent (Supplementary Fig. S3b).

Together, these results demonstrate that our liposome-based mechanical filtration protocol yields *P. falciparum* merozoites with high purity, preserved viability, and preserved invasive potential (Fig. 2 and S3). The ability of these merozoites to invade and complete subsequent intraerythrocytic development underscores the robustness and reproducibility of this isolation method. Encouraged by these results, we extended the approach to *P. vivax* isolates to evaluate its broader applicability.

### Isolation and characterization of viable Plasmodium vivax merozoites.

We assessed whether the workflow developed with *P. falciparum* 3D7 could be applied successfully to *P. vivax*, using clinical isolates obtained from naturally infected patients. To evaluate the integrity of isolated *P. vivax* merozoites, dual labeling was performed using Hoechst to visualize parasite nuclei and antibodies against the *P. vivax* Duffy Binding Protein (PvDBP), a key merozoite surface ligand involved in reticulocyte invasion. Immunofluorescence analysis was conducted on both segmented schizonts prior to filtration and on the merozoite-enriched fractions obtained after 10 FC.

**Figure 3:**
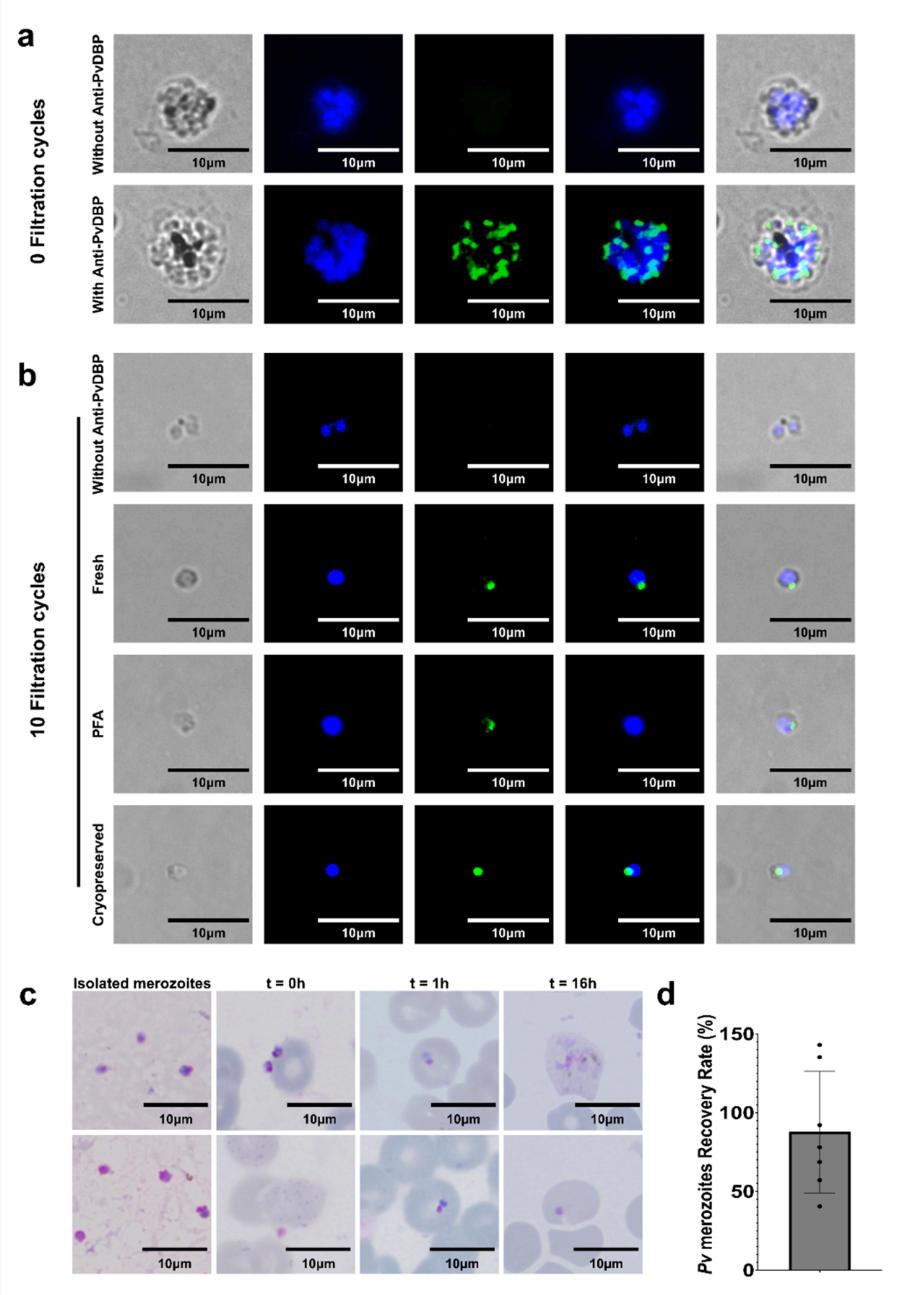
Isolation and functional characterization of *Plasmodium vivax* merozoites. **a**, Representative brightfield and fluorescence images of segmented *P. vivax* schizonts following 3 h treatment with 200 nM ML10 and prior to filtration. Parasites were stained with Hoechst to visualize nuclei and immunolabeled with anti-PvDBP antibodies to detect the Duffy Binding Protein. From left to right: brightfield, Hoechst, PvDBP, and merged images. **b**, Representative brightfield and fluorescence images of isolated *P. vivax* merozoites obtained either from fresh cultures after 3 h treatment with 200 nM ML10 followed by 10 filtration cycles (10 FC), or from fixed and cryopreserved segmented schizonts after 3 h treatment with 200 nM ML10 followed by 15 filtration cycles (15 FC). Merozoites were stained with Hoechst and immunolabeled with anti-PvDBP antibodies. From left to right: brightfield, Hoechst, PvDBP, and merged images. **c,**Representative Giemsa-stained thin blood smears showing **mechanically isolated** *Plasmodium vivax* **merozoites** and their **co-incubation with enriched reticulocytes over time**. Images show isolated merozoites prior to host cell contact, followed by smears prepared at **t = 0 h**, corresponding to the **time of initial contact** between merozoites and reticulocytes. At **t = 1 h**, newly infected reticulocytes containing early ring-stage parasites are observed. At **t = 16 h**, two representative fields illustrate divergent developmental outcomes, with the presence of a **young trophozoite** in one field and a **pyknotic ring form** in the other. Scale bars, 10 μm. **d**, Recovery rate of *P. vivax* merozoites obtained from fresh clinical isolates after 3 h treatment with 200 nM ML10 followed by 10 filtration cycles, expressed as a percentage of the theoretical merozoite yield. Scale bars, 10 μm.

Segmented schizonts displayed clear nuclear staining together with PvDBP localization at the periphery of developing merozoites (Fig. 3a). After 10 FC, the isolated merozoites retained strong PvDBP labeling co-localized with Hoechst-stained nuclei (Fig. 3b). Notably, PvDBP fluorescence was consistently concentrated at one pole of the merozoite, consistent with its expected apical localization in invasion organelles. These observations indicate that both the structural integrity and surface proteins organization are preserved throughout the isolation process.

To further assess the versatility of our approach, segmented schizonts were fixed at the segmented stage using 4% paraformaldehyde and subsequently filtered with the liposome extruder, or alternatively cryopreserved in the presence of trehalose to preserve membrane integrity. Following thawing, filtration (15 FC) of fixed and cryopreserved schizonts yielded morphologically intact merozoites with preserved PvDBP localization, as confirmed by bright-field and immunofluorescence microscopy (Fig. 3b).

The functionality of fresh isolated *P. vivax* merozoites was evaluated using short-term invasion assays with enriched reticulocytes. Merozoites obtained after 10 FC were able to invade reticulocytes efficiently and develop into early trophozoites-stage parasites (Fig. 3c). Newly invaded rings were detected as early as 10 minutes after reticulocyte addition, and parasite development to ring stages was observed up to 16 hours post-invasion (Fig. 3c). However, consistent with the current limitations of *P. vivax in vitro* culture systems, the majority of parasites degenerated beyond the ring stage, and only very limited progression to later trophozoite stages was detected.

Quantification of merozoite recovery revealed a recovery rate of 87.84 ± 38.62% (mean ± SD) relative to the theoretical merozoite yield calculated from segmented schizont counts (Fig. 3d). This yield is comparable to that achieved with *P. falciparum*, confirming the robustness and scalability of the method for *P. vivax*.

Together, these results demonstrate that our adapted isolation workflow preserves *P. vivax* merozoite integrity, antigenicity, and invasion capacity, thereby providing a reliable tool to study this otherwise transient and technically challenging parasite stage.

### Antibody-opsonized Plasmodium vivax merozoites induce NK cell activation.

**Figure 4:**
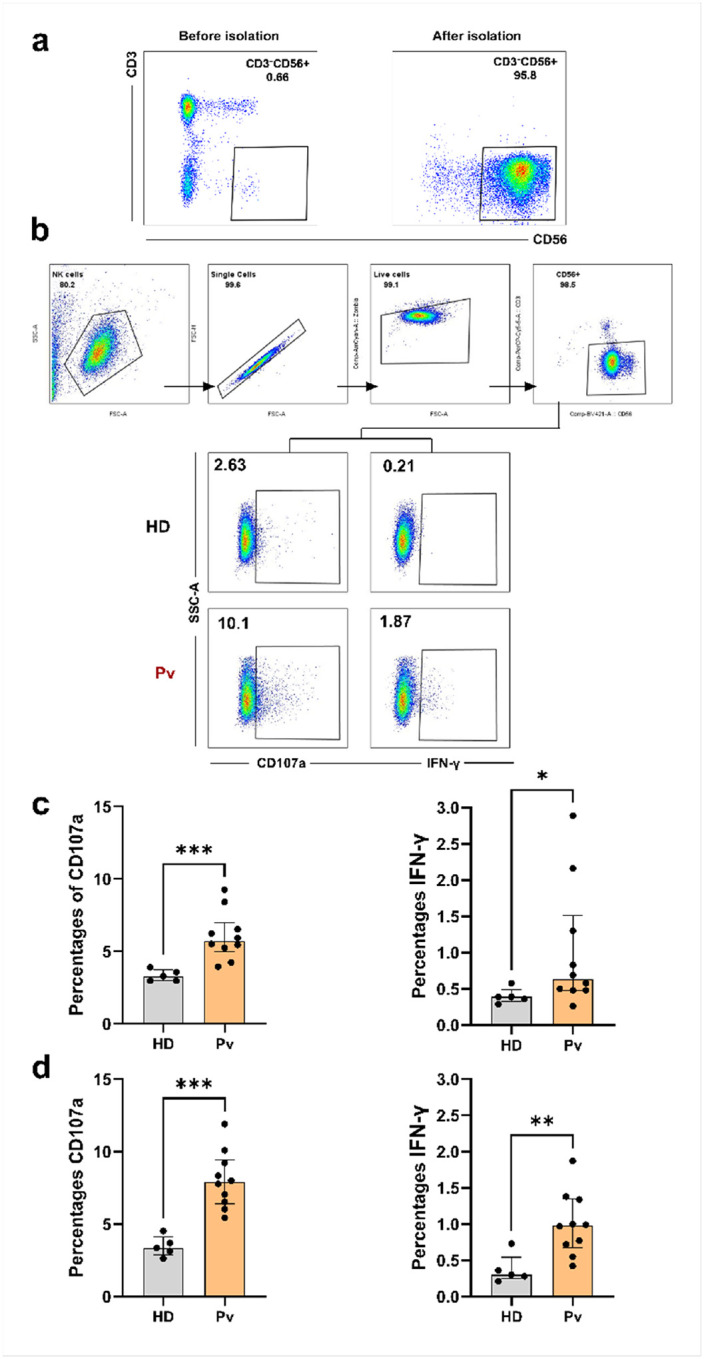
Antibody-dependent activation of NK cells by *Plasmodium vivax* merozoites. **a,** Representative flow cytometry plots of NK cells identified from peripheral blood mononuclear cells (PBMC) or after NK-cell isolation. NK cells were defined as live CD3^−^CD56^+^ lymphocytes **b,** Gating strategy used to identify NK cells and assess activation markers by flow cytometry. NK cells were defined as live CD3−CD56^+^ lymphocytes and analyzed for the expression of CD107a and IFN-γ. **c,** Activation of NK cells following co-incubation with **PFA-fixed** *P. vivax* **merozoites** opsonized with plasma from *P. vivax*-infected individuals (*Pv*+) or malaria-naïve donors (healthy donor (HD). Shown are the percentages of NK cells expressing CD107a and IFN-γ. **d,** Activation of NK cells following co-incubation with **PFA-fixed and cryopreserved** *P. vivax* **merozoites** opsonized with the same plasma from *P. vivax*–infected individuals (*Pv*) or malaria-naïve donors (healthy donor (HD) as in c. Each dot represents one plasma obtained from one donor. Horizontal lines indicate the median and error bars show the interquartile range (IQR). Statistical significance was assessed using a two-tailed Mann–Whitney U test.

To investigate whether *P. vivax* merozoites can trigger NK cell activation in the presence of naturally acquired antibodies, we performed antibody-mediated NK activation assays using purified NK cells from healthy donors. Purity of NK cells (CD3^−^CD56^+^) was routinely assessed by flow cytometry (Fig. 4a). *P. vivax* merozoites that were either PFA-fixed or PFA-fixed and cryopreserved were incubated with heat-inactivated plasma from *P. vivax* infected individuals or malaria-naïve healthy donors. NK cell activation was assessed by flow cytometry using degranulation marker CD107a and intracellular IFN-γ production (Fig. 4b)

When NK cells were incubated with immune complexes originating from merozoites and sera from *P. vivax* seropositive individuals, NK cells displayed a significant increase in activation markers compared with merozoites opsonized with plasma from healthy donors. Both the percentages of CD107a^+^ NK cells (P = 0.0007) and IFN-γ production by NK cells was significantly increased (P = 0.0433), (Fig. 4c).

Next, we determined whether NK cell activation is preserved when using cryopreserved merozoites. Therefore, we repeated the assay using the same source of NK cells, heat inactivated plasma samples but with cryopreserved merozoites. As with fresh merozoites, NK cell degranulation and cytokine production was significantly increased when incubated with immune complexes originating from merozoites with plasma obtained from *P. vivax* seropositive individuals compared to immune complexes originating from merozoites with plasma obtained from healthy donors (P = 0.0007 and P = 0.0047) (Fig. 4d).

These data show that antibody-dependent NK cell activation is similar when using cryopreserved *P. vivax* merozoites, indicating that neither fixation nor cryopreservation compromises assay functionality (Fig. 4d). Taken together, these data show that antibody-opsonized merozoites efficiently trigger NK cell activation, demonstrating that this Fc-dependent functionality can be measured *in vitro* using purified merozoites.

## Discussion

Malaria pathogenesis is driven by blood-stage replication of *Plasmodium* parasites, placing the merozoite at the center of attention for understanding how infection is established and for developing interventions that block parasite replication. However, because the extracellular merozoite is extremely short-lived, studying its biology remains challenging for most *Plasmodium* species particularly *Plasmodium vivax*, which cannot be continuously propagated *in vitro*.

In this study, we successfully established a robust experimental workflow that enables the isolation of viable, synchronous and invasive *P. vivax* merozoites. Moreover, we developed a complementary approach to cryopreserve parasites at the segmented schizont stage, allowing for subsequent merozoite isolation in future experiments. Together, these approaches overcome a long-standing technical barrier in *P. vivax* research by providing both temporal control over parasite egress and sustained access to functional merozoites for downstream analyses.

For the first time, we show that ML10 induces a reversible block of *P. vivax* egress, supporting the notion that this parasite relies on conserved PKG-dependent signaling pathways previously described in *P. falciparum* and *P. knowlesi*^3, 7, 26^. These findings extend the conservation of PKG-mediated egress control to *P. vivax* and provide a practical means to synchronize parasites at the segmented schizont stage. *In vitro*, ML10 treatment increased the proportion of segmented schizonts, thereby significantly enhancing the pool of merozoites that can be recovered from *ex vivo P. vivax* cultures.

A key advance of our study lies in the application of a liposome-based extrusion approach for merozoite isolation. This strategy enables rapid mechanical release of merozoites and markedly improves recovery compared to previously described protocols for *P. falciparum* and *P. knowlesi*^8, 15, 17^. The high efficiency of this approach makes it suitable for isolating *P. vivax* merozoites, even from limited quantities of parasites providing enough material for downstream studies on this short-lived extracellular stage. Importantly, the speed of this process minimizes the time merozoites spend extracellularly during isolation, preserving their viability and invasive capacity.

Previous approaches to isolate *P. vivax* merozoites have been limited by low recovery and contamination with other parasite stages^8, 15^. A widely referenced method developed for *P. falciparum*^8^ relies on a single filtration through a 1.2 μm membrane and typically yields ~ 4 × 10^8^ merozoites from 100 mL of parasite culture. Based on estimated RBC counts and merozoite numbers per segmented schizont, this corresponds to a recovery rate of approximately 0.14%. While such yields are sufficient for *P. falciparum* or *P. knowlesi*, which can be readily amplified *in vitro*, they are incompatible with *ex vivo P. vivax* cultures that are poorly synchronized and available in limited quantities. Under these conditions, obtaining the number of merozoites required for functional immunological assays would necessitate parasite inputs and therefore blood volumes far beyond what can reasonably be obtained from clinical samples. This practical constraint has likely contributed to the scarcity of functional studies targeting the *P. vivax* merozoite stage.

In this study, we also demonstrate that a single filtration cycle using 2 μm or 5 μm membranes, as described in previous studies, is insufficient to achieve true merozoite isolation^28^. Under such conditions, many merozoites remain trapped within the parasitophorous vacuole membrane or form aggregates, and a fraction of uninfected and infected RBCs can pass through the filter, potentially biasing downstream analyses. Unexpectedly, the use of a 5 μm membrane enabled the recovery of intact parasites devoid of their host red blood cells, thereby mimicking a pitting-like phenomenon. Although this observation was not further explored in the present study, the approach described here may provide a cost-effective and accessible alternative for *in vitro* investigation of parasite pitting, compared with more complex microfluidic systems currently employed^29^.

In contrast, our approach achieves recovery rates exceeding 75% for *P. vivax* merozoites while markedly reducing contamination by residual RBC and eliminating other parasite stages. This substantial increase in efficiency represents a qualitative shift that makes functional and immunological analyses feasible for *P. vivax*.

The short duration of the process is essential, as it preserves merozoite viability and invasive capacity known to decline drastically within 15 minutes after egress in *P. falciparum*. Using our optimized method, *P. vivax* and *P. falciparum* merozoites remain capable of invading reticulocytes and progressing to the ring stage, or complete blood stage cycle for *P. falciparum*, confirming their biological functionality.

Another major advance of our study is the development of a cryopreservation strategy for segmented schizonts using a trehalose-based solution. This approach using trehalose as extracellular cryoprotectant preserves membrane integrity and the overall organization of merozoite surface structures during storage^30^. Remarkably, segmented schizonts that were fixed with 4% paraformaldehyde and cryopreserved in trehalose could later be filtered through the liposome extruder to yield morphologically intact merozoites with recovery rates comparable as fresh fixed schizonts. The ability to fix and cryopreserve segmented schizonts while preserving essential structural and molecular features represents a significant methodological advance. It opens the possibility of collecting and storing *P. vivax* schizont samples directly in endemic regions and distributing them to research laboratories worldwide. This advancement could facilitate global efforts to characterize the biology of the *P. vivax* merozoite stage and accelerate the development of vaccines or invasion-blocking therapies targeting this elusive parasite.

Importantly, our results demonstrate that *P. vivax* merozoites opsonized with sera from infected patients can stimulate NK cell degranulation and cytokine production. We demonstrate as proof of principle that our merozoite purification strategy will enable us to evaluate further *P. vivax* directed NK-cell activation and other antibody effector functions in future studies. This previously underexplored effector mechanism highlights the functional relevance of naturally acquired antibodies against merozoites. Indeed, in the context of *P. falciparum*, Ab-dependent NK cell activation was associated with successful control of parasitemia after experimental malaria challenge, and was associated with lower risk of clinical episodes of malaria in naturally infected children^31^. Notably, FcγRIIIa binding has been identified as a potential immune correlate of protection in controlled human malaria infection studies^32^. As FcγRIIIa (CD16a) is the principal activating Fc receptor mediating antibody-dependent NK cell cytotoxicity, our findings provide a mechanistic framework linking antibody Fc functionality to NK cell activation in the context of *P. vivax* merozoites. Consistent with this framework, a recent longitudinal cohort study in children identified antibody Fc-mediated functions, including Fcγ receptor engagement and complement fixation against multiple *P. vivax* antigens, as correlates of protection from clinical malaria. Importantly, antibodies targeting merozoite-associated antigens and exhibiting strong FcγR binding profiles were among those most strongly associated with reduced disease risk, supporting the biological relevance of Fc-dependent effector mechanisms in naturally acquired immunity^33^. Given that anti-merozoite antibodies can inhibit or delay red blood cell invasion, such antibody-mediated effects may extend the extracellular exposure time of merozoites, thereby creating a window for Fc receptor-mediated NK cell activation. These findings further emphasize the importance of obtaining highly pure and intact merozoites, as enabled by our optimized isolation method, which provides sufficient material to perform functional assays that were previously limited for *P. vivax*.

In conclusion, by overcoming long-standing technical barriers, this work opens *P. vivax* merozoite biology to functional, immunological, and comparative investigation, enabling questions that were previously inaccessible for this globally important malaria parasite.

## Supplementary Material

Supplementary Files

This is a list of supplementary files associated with this preprint. Click to download.

- SupplementaryFigures.docx
- Supplementaryvideo1.mp4
